# Nurses’ Role in Patient Education for Managing Inflammatory Joint Diseases: Insights from a Cross-Sectional Survey in Bulgarian Rheumatology Clinics

**DOI:** 10.3390/healthcare13192516

**Published:** 2025-10-03

**Authors:** Stefka Stoilova, Stanislava Popova-Belova, Mariela Geneva-Popova

**Affiliations:** 1 Department of Healthcare Management, Faculty of Public Health, Medical University—Plovdiv, 4002 Plovdiv, Bulgaria; 2 Department of Propaedeutics of Internal Diseases, Faculty of Medicine, Medical University—Plovdiv, 4002 Plovdiv, Bulgaria; stanislava.popova@mu-plovdiv.bg (S.P.-B.); mariela.geneva@mu-plovdiv.bg (M.G.-P.)

**Keywords:** biologic disease-modifying antirheumatic drugs (bDMARDs), health behaviors, holistic care, inflammatory joint diseases, nurses’ role, patient activation, patient education, self-injection skills

## Abstract

**Background**: Nurses play a central role in the management of inflammatory joint diseases (IJD), of which the success depends on patient adherence to treatment, self-monitoring, timely detection of adverse drug reactions (ADRs), and adopting a healthy lifestyle. This study sought to examine the opinions of patients with IJD regarding the educational and supportive contributions of nurses. **Methods**: The research is based on a cross-sectional survey of patients with IJD treated with biologic disease-modifying antirheumatic drugs (bDMARDs) in two rheumatology clinics in Plovdiv, Bulgaria, from the beginning of August 2024 to the end of January 2025. The group included patients of three diagnoses: (1) rheumatoid arthritis (RA), (2) psoriatic arthritis (PsA), and (3) axial spondyloarthritis (axSpA). **Results**: Regardless of the diagnosis, and after adjusting for covariates, patients rated the roles of nurses in disease treatment and management, the acquisition of self-injection skills for bDMARDs, the implementation of a healthy lifestyle, and the maintenance of psychological well-being at the higher end of the 0 to 4 scale. However, the axSpA patients were less affirmative in their responses compared to the RA and PsA patients. In the RA and PsA groups, the working patients were associated with the lowest ratings, followed by retirees with disability. **Conclusions**: Our findings indicate that nurse-led education in patient self-management skills is greatly appreciated by patients with IJD. Further developments in specialized training programs tailored to the specific needs of different diagnoses and in consideration of patients’ social status will lead to increased patient satisfaction and a better overall quality of life.

## 1. Introduction

Rheumatoid arthritis (RA), axial spondyloarthritis (axSpA), and psoriatic arthritis (PsA) are some of the most common inflammatory joint diseases (IJDs), associated with severe physical, mental, and social problems. The introduction of biologic disease-modifying antirheumatic drug therapies (bDMARDs) to treat these diseases has fundamentally altered their progression and patient outcomes. However, the effectiveness of therapies depends heavily on active patient involvement and the quality of healthcare [1,2,3]. In this context, the nurse’s role is vital, as they administer treatment, educate, monitor, and provide psychosocial support to the patient. Management of IJDs requires more than pharmacotherapy. It requires structured care, health literacy, and ongoing support [4]. Nurses are increasingly recognized as key figures in providing patient-centered education and psychosocial care [5,6]. In line with the requirements of national legislation, nurses provide patient-centered care tailored to the specific needs and characteristics of each patient. At the same time, nurses must adapt to the changing preferences, needs, and values of patients [7].

The purpose of this article is to explore the opinions of IJD patients of three diagnoses, RA, PsA, and axSpA, on the competence and efficacy of nurses in different domains of patient education, including disease and treatment informedness, self-injection training for bDMARDs, coaching in healthy eating, physical activities, abstinence from tobacco and alcohol, and emotional and psychological support.

The application of biological treatment in rheumatology is constantly expanding. The European Alliance of Rheumatology Associations (EULAR) states that the treatment of rheumatic diseases should be carried out by a multidisciplinary team (MDT) [8], where nurses are a mandatory component [9]. As members of the MDT, nurses are ideally positioned to explore the needs and preferences of patients with IJDs and implement appropriate training and support towards improved patient quality of life and mental health.

### The Role of Nurses in the Holistic Care of Patients with IJDs

According to EULAR, the nurse’s role in the management of chronic inflammatory arthritis includes providing education activities and support that ensure comprehensive patient care. Specifically, the EULAR recommendations outline key areas of training and support, such as: educating patients according to their needs and preferences; monitoring and counseling on possible adverse events; providing psycho-emotional support; maintaining effective communication; and providing support for self-management and adherence to treatment [10].

Several studies have reported on rheumatology nurses operating telephone advice lines, offering self-management support and education, conducting patient counseling, and mediating communication with other members of the medical team [11,12,13,14,15].

Nurses play an important role in disease treatment and monitoring, and they assist in the management of comorbidities [16,17,18]. For patients on biologic disease-modifying antirheumatic drugs (bDMARDs), nurses are expected to educate them on the mechanism of action, route of administration (e.g., subcutaneous injection), the importance of adherence to treatment, and monitoring for adverse reactions. Effective administration of biologic therapies requires nurses to coordinate and monitor the therapy, conduct surveillance, and manage drug reactions and side effects associated with bDMARDs’ therapy [19]. This includes participating in the administration of intravenous medications or teaching patients how to self-administer their medications [20], which leads to improved patient adherence to therapy, enhanced quality of life, and better self-management [21].

Nursing support for patients with IJDs on biologic therapy can also be manifested in the provision of guidance on healthy lifestyles, weight control, and follow-up for treatment adherence [22]. At the same time, nurses should manage patients’ psychological problems [10] by offering emotional support to cope with the stress and fears associated with the administration of biological therapy [23].

An effective approach to biologic treatment may be the provision of comprehensive, holistic care by nurses aimed at improved disease management, quality of life, and outcomes in patients with IJD [24,25]. However, other literature has shown that nursing practice generally lacks a holistic, systematic, and comprehensive approach to providing education and support for these patients [26].

In Bulgaria, nurses earn their qualifications through specialized bachelor’s programs that meet the European Union’s professional standards. It is expected that nurses specializing in the care of patients with IJD should be equipped to coach these patients in managing their disease and treatment, as well as in adopting healthy lifestyle habits that can enhance treatment outcomes. However, there is a lack of research regarding the role of nurses in training patients with IJD on bDMARD therapy to develop independent lifestyle skills.

In light of this, our team aimed to investigate how patients with IJD on bDMARD therapy perceive the role of nurses in their disease treatment and management. We deemed that the findings will be valuable not only for understanding the current state of care but also for informing future modifications and enhancements to the education of rheumatology nurses.

Specifically, we focused on the following aspects of nurses’ roles: (1) Patient education about the specifics of the disease, treatment, and medical exams; (2) Training patients to self-inject bDMARDs and cope with adverse drug reactions; (3) Providing coaching and support in patients’ adopting healthy eating habits, physical activities, control over tobacco and alcohol consumption, and maintaining psychological and emotional well-being.

## 2. Materials and Methods

This research involved a cross-sectional survey with patients with inflammatory joint disease (IJD), involving three diagnoses: (1) rheumatoid arthritis (RA), (2) psoriatic arthritis (PsA), and (3) axial spondyloarthritis (axSpA), treated in rheumatology clinics in Bulgaria.

The study complied with the requirements of the World Medical Association Declaration of Helsinki (1964) and its 2000 modification (Edinburgh) regarding the involvement of human subjects. The research design and procedures were approved by the scientific ethics board at Medical University—Plovdiv, Bulgaria (approval code No. P-17188, approval date: 24 July 2024).

The patients were recruited over a six-month period, from the beginning of August 2024 to the end of January 2025, at two rheumatology clinics in Plovdiv, the second largest city in Bulgaria, located in the south-central region. During this time, all patients who visited the clinics for routine check-ups were asked to complete a paper survey. The patients were entitled to decline participation without any reflection on their treatment. All patients included in the study provided written informed consent for voluntary participation, as well as for the publication of data in scholarly journals.

The participants in the study met the following inclusion criteria: (1) A confirmed diagnosis of rheumatoid arthritis, psoriatic arthritis, and ankylosing spondylitis according to the European League Against Rheumatism (EULAR); (2) Age over 18 years; (3) Currently undergoing treatment with biologic disease-modifying antirheumatic drugs (bDMARDs); (4) In good mental health and demonstrating adequate understanding; (5) Provided consent to participate in the survey by signing an informed consent form.

The survey combined standardized scales and researcher-created items. The standardized part incorporated the five-dimensional EuroQol questionnaire [EQ-5D-3L] and the EuroQol Visual Analogue Scale (EQ-VAS). The present article did not use data from these scales and was entirely based on researcher-developed items that define nurses’ competence to act as educators and trainers, including teaching self-management skills related to patients’ diseases and treatment specifics, self-injection of biologic disease-modifying antirheumatic drugs (bDMARDs), managing adverse drug reactions, and promoting healthier eating and lifestyle habits. These items were developed by our team in close consultation with rheumatology doctors and nurses. We took the following steps in developing the questionnaires:

1. Formulating the main constructs of the survey
2. Creating items for each construct and determining their type (yes/no, multiple choice).
3. Editing the items and adding explanatory text where necessary.
4. Pilot testing the first draft with 10 patients, whose responses were not included in the data pool.
5. Making further edits and clarifications based on the insights gained through the pilot test.

The responses were measured on a Likert scale of five options: 0 = “definitely no,” 1 = “rather no,” 2 = “neutral,” 3 = “rather yes,” and 4 = “definitely yes.” For the purpose of the statistical analysis, the responses were treated as a continuous scale, allowing for parametric comparisons between the three diagnoses and control over confounding variables through an analysis of covariates.

We conducted Cronbach’s alpha reliability analysis on the researcher-developed items, addressing nurses’ role in patient self-management skills. The results showed a good overall internal consistency with the following statistics: Cronbach’s alpha on raw items = 0.813, 95% lower CI = 0.783; Cronbach’s alpha on standardized items = 0.773, 95% lower CI = 0.737.

### 2.1. Statistical Analysis

The statistical software IBM SPSS Statistics for Windows, Version 28.0 (Armonk, NY, USA: IBM Corp.) was used to analyze the data. The Shapiro–Wilk test was applied to continuous variables in the pre-screening stage to determine the shape of the distributions. The data was described with the mean values and standard deviations (SD) when normality was observed. For each family of related dependent variables (e.g., items reflecting informedness and those reflecting health behaviors), we conducted a multivariate analysis of covariance (MANCOVA) including age, gender, and social class as covariates. When the multivariate test was significant, we performed follow-up univariate ANCOVAs for each dependent variable, with diagnosis entered as a fixed factor and the same covariates included. Post hoc pairwise group comparisons were adjusted using the Bonferroni method.

When normality was not observed, the median and interquartile range (IQR) were used to describe the data. Comparisons between diagnostic groups were performed through the Kruskal–Wallis test with Bonferroni paired comparisons. Categorical data was presented as numbers and percentages, and associations were examined through the chi-square test, followed by z-tests with Bonferroni adjustments. Statistical significance was accepted at a Type I error of 5% (*p* < 0.05).

Questions that allowed multiple responses were entered in separate columns with binary coding. To control for familywise Type-I error, we applied Bonferroni adjustment to the accepted level of significance (alpha = 0.05), taking into account the number of binary variables.

#### Ruling out Differences Related to Clinic of Treatment

Before conducting the statistical analysis, we compared patients’ ratings on nurses’ roles in the key areas of interest, including informedness about their diagnoses, treatment specifics, and examinations; self-injection training; healthy living and psychological well-being; and overall quality of care. We utilized both *t*-tests for independent samples and Mann–Whitney U tests. Both methods produced similar results, indicating no significant differences between the two clinics, as follows: informedness about the disease (*p* = 0.068 *t*-test; *p* = 0. 053 Mann–Whitney U); informedness about treatment specifics (*p* = 0.287 *t*-test; *p* = 0.152 Mann–Whitney U); informedness about examinations and tests (*p* = 0.266 *t*-test; *p* = 0.160 Mann–Whitney U); self-injection training (*p* = 0.744 *t*-test; *p* = 0.155 Mann–Whitney U test); healthy eating (*p* = 0.325 *t*-test; *p* = 0.315 Mann–Whitney U test); physical activity (*p* = 0.491 *t*-test; *p* = 0.600 Mann–Whitney U test); psychological well-being (*p* = 0.259 *t*-test; *p* = 0.061 Mann–Whitney U test); overall quality of care (*p* = 0.075 *t*-test; *p* = 0.053 Mann–Whitney U test).

## 3. Results

### 3.1. Background Information About the Patients

**Figure 1:**
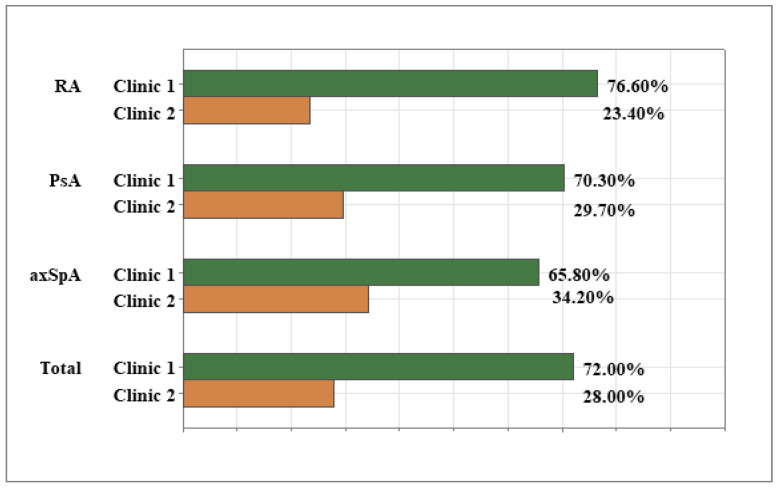
Patient distribution between clinics and diagnoses.

The current study included a total of 261 patients with inflammatory joint disease (IJD). Of them, 124 (47.50%) were diagnosed with rheumatoid arthritis (RA); 64 (24.50%) had psoriatic arthritis (PsA), and 73 (27.90%) had axial spondyloarthritis (axSpA). The patients, who came from two rheumatology clinics in Plovdiv, Bulgaria, were distributed between clinics and diagnoses, as shown in Figure 1. The majority of them (72.00%) were treated at Clinic 1, with a similar distribution of diagnoses between the two clinics (Pearson χ^2^ = 2.81, df 2, *p* = 0.245).

The diagnosis groups differed significantly in median age (*p* < 0.001), with the highest median age in the RA group and the lowest in the axSpA group. The gender distribution also showed significant group differences (*p* < 0.001), with the majority of RA patients being women, an almost equal male-female distribution in the PsA group, and a dominance of male patients in the axSpA group. The axSpA group had the highest proportion of working patients and the lowest proportion of retirees. Working patients also dominated in the PsA group, whereas the RA patients were rather similarly distributed among working, retirees, and disability pensioners. The median time since diagnosis was similar in the three groups (13–14 years), and the median count of visits per year to a rheumatology specialist was the same (2 counts). The majority of the patients (total *n* = 228), regardless of the diagnosis, were treated with injectable bDMARDs with a median duration of treatment between 5.50 and 7 years (Table 1).

### 3.2. Nursing Support in Enhancing Patients’ Informedness About Inflammatory Joint Disease and Treatment

Patients’ ratings of nurses’ role in raising awareness of the disease did not differ significantly among the three diagnoses and were not significantly associated with age (*p* = 0.675), gender (*p* = 0.469), or social status (*p* = 0.397) in the adjusted model. Overall, patients’ evaluations of nurses’ contributions to their understanding of the disease and treatment were very positive across diagnoses. Mean ratings (on a 0–4 scale) were near the maximum in the rheumatoid arthritis (RA) and psoriatic arthritis (PsA) groups, with slightly lower ratings in the axial spondyloarthritis (axSpA) group (Table 2).

### 3.3. Nurses’ Role in Training Patients to Self-Inject bDMARDs

**Figure 2:**
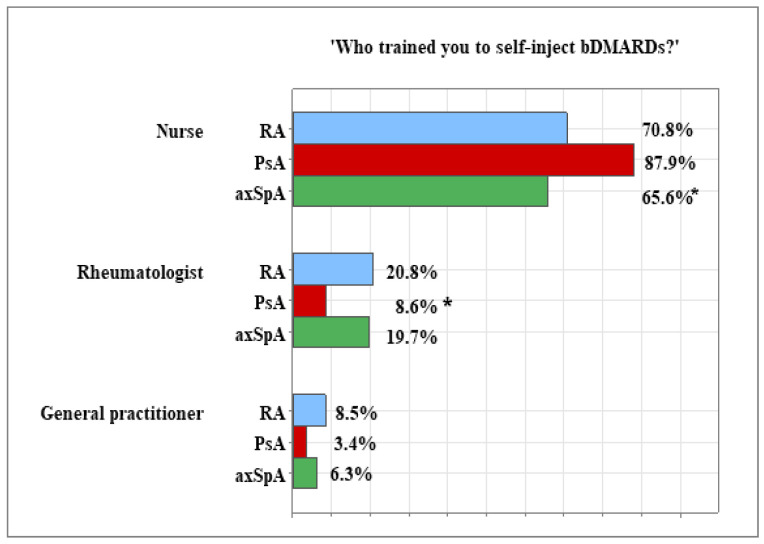
Distribution of patients’ responses to the question ‘Who trained you to self-inject bDMARDs?’ across diagnoses. *—Significant difference at *p* < 0.05.

Of the 261 patients with inflammatory joint disease, 228 were receiving injectable bDMARDs treatment. Among these, as previously mentioned, 106 were with RA, 58 were with PsA, and 64 had axSpA. The majority of these patients were trained to self-inject the bDMARDs by a nurse: 71.70% (*n* = 76) of the RA patients, 87.90% (*n* = 51) of the PsA patients, and 67.20% (*n* = 43) of those with axSpA. The association with diagnosis was significant, with the PsA group showing the highest proportion of patients trained by a nurse compared to the other two groups (*p* = 0.019). Conversely, a significantly smaller proportion of patients in the PsA group were trained by a rheumatology doctor, with 8.60% (*n* = 5) compared to 18.90% (*n* = 20) of the RA patients and 25.00% (*n* = 16) of the axSpA patients (*p* = 0.013). A small percentage of patients reported being trained by a general practitioner: 8.50% (*n* = 9) of the RA patients, 3.40% (*n* = 2) of the PsA patients, and 6.30% (*n* = 4) of those with axSpA. Only two patients, one with RA (0.90%) and one with axSpA (1.60%), indicated they were trained by someone else, such as a family member or a friend (Figure 2).

An ANCOVA was conducted with diagnosis as a fixed factor and training provider (nurse, rheumatology doctor, and general practitioner) as a covariate. The results revealed a significant effect for the training provider (F = 26.26 (2, 224), *p* < 0.001). Patients trained by a nurse expressed a significantly greater trust in nurses’ ability to teach self-injection skills (mean = 3.95, SD = 0.22) than those trained by a rheumatologist (mean = 3.56, SD = 0.81) or a general practitioner (mean = 3.54, SD = 1.10), F = 26.26 (2, 224), *p* < 0.001). After adjusting for the training provider, estimated marginal means (EMMs) indicated no significant differences in confidence across the three diagnostic groups (*p* = 0.710). Patients’ ratings were in the upper part of the scale, with the highest rating provided by the RA patients and the lowest by the PsA patients (Table 3).

**Figure 3:**
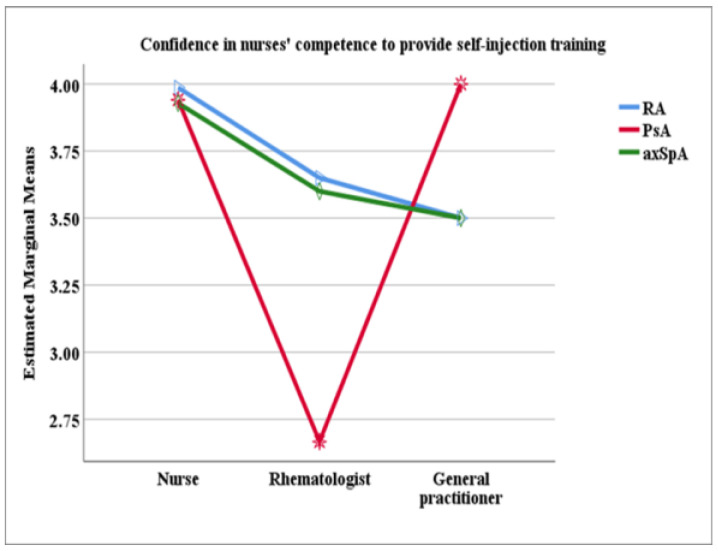
Plot of the interaction between diagnoses and providers of training in relation to patients’ confidence in nurses’ competence to deliver self-injection training.

Figure 3 presents the estimated marginal means (EMMs) for patients’ ratings of nurses’ competence to deliver self-injection training, stratified by diagnosis and by the specialist who provided the training. The interaction plot is primarily descriptive due to the small number of observations in some subcategories. Nonetheless, a clear trend is evident for the RA and axSpA groups: patients trained by a nurse expressed the highest trust in nurses’ competence, followed by those trained by a rheumatologist, and the lowest trust was reported by those trained by general practitioners. In contrast, among PsA patients, those trained by a rheumatologist showed the lowest trust in nurses’ competence, whereas those trained by a general practitioner reported the highest confidence in nurses’ competence.

### 3.4. Nurses’ Role in Educating Patients with IJD to Adopt Healthy Living Habits and Maintain Psychological Well-Being

The results presented in this subsection include the entire sample of 261 patients. Statistical analysis indicated no significant associations between the covariates age (*p* = 0.700), gender (*p* = 0.157), and social status (*p* = 0.100) in relation to patients’ responses regarding nursing support for healthy living habits and psychological well-being. Univariate comparisons among the three diagnoses did not show significant differences in patients’ ratings of the nurses’ role in guiding them toward adopting healthier lifestyles. The mean ratings concerning education on healthy eating, alcohol and tobacco use, promotion of a healthier culture, and suitable physical activities fell in the range between “rather yes” and “definitely yes” across all patient groups, with no significant differences observed. Nurses’ support for psychological and emotional well-being received the highest ratings, with mean scores close to “definitely yes.” Descriptively, patients with axSpA provided the lowest ratings on all three questions (Table 4).

### 3.5. Patients’ Preparedness in the Event of Adverse Drug Reactions (ADRs)

Another aspect of effective therapy involves educating patients to recognize adverse drug reactions (ADRs) and, if needed, to contact a member of the treatment team for assistance and advice. The totals for diagnosis groups exceed 100% because patients provided more than one response. The alpha level for the chi-square tests was adjusted to 0.016 (Bonferroni 0.05/3) to control the familywise Type-I error across the three related binary items from the same question.

Our results showed that over 95% of all patients, regardless of the diagnosis, were informed about the potential ADRs of the treatment, namely 97.30% of the RA patients, 98.50% of the PsA patients, and 95.80% of the patients with axSpA.

If patients experienced AEs, the majority would choose to contact a rheumatology doctor, with no significant differences among the diagnoses (χ^2^ = 4.415, *p* = 0.621), including 94.40% (*n* = 117) of the RA patients, 92.20% (*n* = 59) of the PsA patients, and 91.80% (*n* = 67) of the patients with axSpA. The patients who indicated reaching out to a nurse constituted 41.10% (*n* = 51) of the RA group, 34.40% (*n* = 22) of the PsA group, and 24.70% (*n* = 18) of the axSpA group. The observed differences were descriptive without statistical significance (χ^2^ = 9.089, *p* = 0.169).

**Figure 4:**
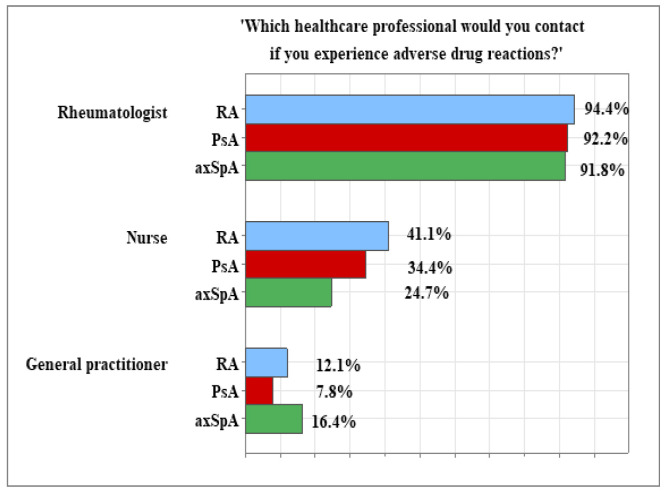
Distribution of patients’ responses to the question ‘Which healthcare professional would you contact if you experience adverse drug reactions?’.

In all groups, the proportions of patients who would contact their general practitioners were small, amounting to 12.10% (*n* = 15) in RA patients, 7.80% (*n* = 5) in PsA patients, and 16.40% in axSpA patients, with no significant differences among the diagnoses (χ^2^ = 7.755, *p* = 0.101). Figure 4 presents patients’ preferences for a specialist to contact in case of adverse drug reactions.

### 3.6. Nursing Role in Enhancing the Overall Quality of Care for Patients with IJD

The patients were asked to assess nurses’ contribution to the overall quality of care for patients with IJD. An ANCOVA analysis with diagnosis as a fixed factor, and age, gender and social class as covariates showed three significant effects: diagnosis (*p* = 0.019), social status (*p* = 0.028), and the interaction between them (*p* = 0.039). Patients’ ratings were not significantly associated with their age (*p* = 0.757) and gender (*p* = 0.147).

The RA and PsA patients rated nurses’ contributions to quality care toward the high end of the scale, whereas the axSpA patients were less affirmative. The difference in ratings between the RA patients and the axSpA patients was significant in Bonferroni-adjusted post hoc comparisons (*p* = 0.015).

By social status, the disability pensioners provided the highest ratings, followed by the working patients, with retirees providing lower ratings overall. The difference between the disability pensioners and the retirees was significant (Bonferroni *p* = 0.030).

**Figure 5:**
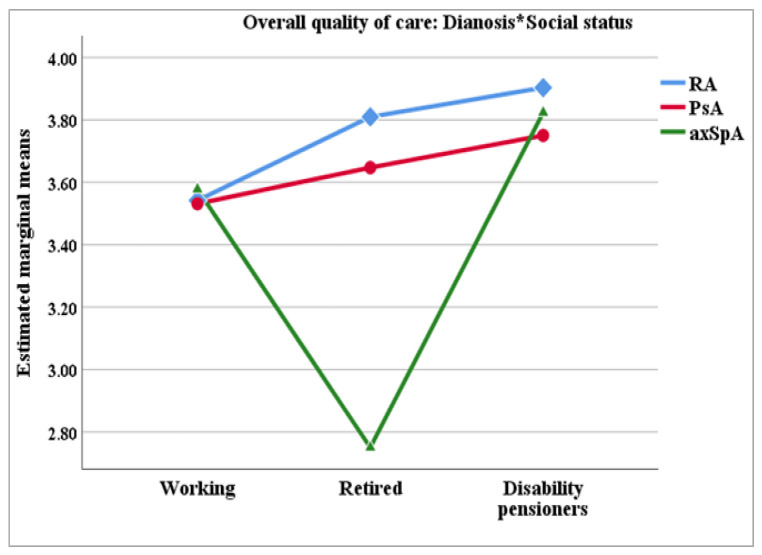
Line plot of the interaction effect between diagnosis and social status regarding patients’ ratings on overall quality of care. *: interaction.

The interaction between diagnosis and social status revealed a slightly different pattern from the one observed in the main effects. The significant differences between the social status groups varied for each diagnosis, as is shown in Table 5. For RA and PsA patients, the trend showed working patients providing the lowest ratings, followed by retirees, with disability pensioners giving the highest ratings. However, for axSpA patients, the lowest ratings were provided by retirees. Across diagnoses and social status, the highest ratings for all three diagnoses were provided by the disability pensioners (Figure 5).

## 4. Discussion

Nurses are in a unique position to assist patients with inflammatory joint diseases in acquiring self-management skills, such as symptom monitoring, medication adherence, education in self-injection of bDMARDs, healthy living habits, and psychological well-being. The present study compared insights from patients with three IJD diagnoses, including RA, PsA, and axSpA, regarding their opinions on the role of nurses in patient education for disease and treatment management, self-injection of bDMARDs, and encouragement to adopt healthy lifestyles [19].

The groups differed in terms of age, gender, and social status, and those variables served as covariates in the statistical analysis. The youngest in age were the axSpA patients, followed by the PsA and the RA patients. Women were dominant in the RA group, whereas men dominated among the axSpA patients. Similar observations were reported in other studies [27,28].

The PsA group showed more even distribution by gender [29]. The RA group had an equal distribution of working, retired, and disability pensioners, whereas in the other two groups, the working patients were the majority. These demographic differences carry social implications, as they identify diagnoses with certain social profiles. Recognizing them can lead to customized healthcare interventions that accommodate the specifics of each demographic and further enhance the quality of life for patients with various forms of inflammatory arthritis [30].

Regardless of their diagnosis, patients provided high ratings for nurses’ role in educating them about disease and treatment specifics. The lack of significant variation across diagnostic groups in our study suggests that arthritis patients valued nurses’ interventions and support in managing their conditions. However, despite the lack of significant differences, the patients with axial spondyloarthritis (axSpA) provided the lowest ratings. This may be attributed to the unique needs of axSpA patients, whose diagnosis might not necessitate a strong reliance on nursing support. Research has highlighted the differing educational needs of axSpA patients compared to those with psoriatic arthritis (PsA) [31]. Nevertheless, this issue presents an opportunity for further investigation and for enhancing the role of nurses in axSpA care, especially in the domains of patient education and self-management support [32].

An important aspect of patients’ quality of life is their flexibility and independence. Since in our sample, 87.35% of the patients were treated with injectable bDMARDs, it was important to train them in self-injection skills that would lead to more flexible schedules and an enhanced sense of self-reliance. This type of training is provided by the medical staff involved in the treatment and management of patients’ conditions, including nurses, rheumatologists, and general practitioners. According to patient reports, the majority received training from a nurse, with the highest proportion in the PsA group and the lowest in the axSpA group. However, a significantly smaller percentage of patients in the PsA group received training from a rheumatology doctor compared to those with RA and axSpA. The variations in providers of self-injection training across diagnoses may be due to policy differences and practical considerations among the medical teams that were involved in the patient treatment [33].

Patients’ confidence in the provider’s competence for self-injection training was significantly associated with who they were trained by [34]. Patients trained by a nurse expressed greater trust in nurses’ ability to provide this type of service compared to those trained by a rheumatology doctor or a general practitioner. Overall, patients demonstrated a very high level of trust in nurses’ competence across the three diagnoses. However, on the descriptive level, the PsA patients showed a different pattern. They gave the lowest ratings, despite the fact that a nurse conducted training for the highest proportion of them. Moreover, PsA patients trained by a rheumatologist exhibited the least trust in nurses’ competence, while those trained by a general practitioner expressed the greatest confidence in nurses’ abilities.

The overall high ratings for nurses’ ability to train patients in self-injection skills endorse the important role of nursing staff in enhancing patients’ treatment efficiency and well-being. However, the variations among diagnoses, while mainly descriptive, point to specific areas that require further exploration and development in healthcare practices concerning trainer-patient education tailored to each diagnosis. Why did patients with PsA deviate from the general trend by expressing the lowest level of confidence in the training abilities of nurses, despite the fact that they received the majority of their training from a nurse? Hypothetical answers may be related to the quality of nurse–patient communication during the training process, the specifics of the diagnosis, the organization of the training, and other factors. A Chinese study has shown that PsA patients’ satisfaction with the treatment depends on their individual characteristics and personal experiences [35]. Other studies have shown that PsA patients’ attitudes toward the treatment are influenced by the characteristics of the drugs themselves and may evolve with treatment experience [36,37]. Awareness of these factors can lead to more efficient training methods, enhanced patient confidence, and improved communication. Our findings are consistent with a randomized trial demonstrating the beneficial effect of a nurse-led intervention to increase patient safety skills in the administration of bDMARDs [38]. Patient support programs (PSPs) that educate patients about the injection process, the disease, and the treatment can improve their adherence to and satisfaction with the care [39,40].

In our study, patients’ appreciation of nurses’ contribution to their overall well-being was supported by their high ratings of the nurses’ role in guiding them toward adopting healthier lifestyles. Nurses’ support for patients’ psychological and emotional well-being received the highest ratings, with mean scores approaching the high end of the scale. Patients’ evaluations of nurses’ role in providing guidance on healthy eating, alcohol and tobacco control, and suitable physical activities were affirmative across diagnoses, with no significant differences in ratings. Our results support the findings of other studies that have reported that nurses play an important role in patients’ adherence to the treatment, disease-management skills, and overall satisfaction with the quality of care [20,41,42,43].

Consistent with prior work [20], patients with axSpA provided the lowest ratings. They also deviated from the observed trend in the overall assessment of the nurses’ role for quality of care. While the RA and PsA patients rated nurses’ contributions at the high end of the scale, the axSpA patients were less affirmative.

This difference in perception may reflect several factors. Because our study did not measure these influences, any explanation is speculative. One possibility is that the specific needs of axSpA patients are not fully addressed by current models of care [44]. Such patients often experience inflammatory back pain (IBP), prompting them to seek help from GPs, osteopaths, pain specialists, chiropractors, and physiotherapists. When extra-articular symptoms (psoriasis, inflammatory bowel disease, or uveitis) also manifest, patients with axSpA are referred to a dermatologist, gastroenterologist, or ophthalmologist [45].

In their study, Crossland et al. described a multidisciplinary treatment of axSpA patients, involving physiotherapists, rheumatologists, and occupational therapists, as well as social workers, nurses, and psychologists [46]. Consequently, nurses do not occupy primary leadership positions in the MDE for patients with axSpA. The education of patients with axSpA should be individually tailored based on an established trusting relationship between the patient and the healthcare professional, and multidisciplinary and interdisciplinary collaboration between healthcare professionals should be established [47]. Lower scores from patients with axSpA may indicate a need for further education and training for nurses on specific aspects of axSpA care. Further research is necessary to explore the relationship between the specific needs of patients with axSpA and the care that nurses can offer, with a strong focus on the effectiveness of nurse-led interventions [48].

In contrast to our results, a study by Molto et al. provided confirming evidence for the effectiveness of nurse-led self-management for disease activity in patients with axSpA. The research examined the effect of three main interventions: (1) educating patients to calculate and record patient-reported outcomes (PROs); (2) nurse evaluations of patients’ need for intensive physical therapy; and (3) patient education on the importance of physical activity, the use of nonsteroidal anti-inflammatory drugs (NSAIDs), and the reduction in tobacco use [49].

In our study, social status was a significant factor for patients’ level of satisfaction with the care provided by nurses. The highest ratings were associated with the disability pensioners, followed by the working patients, with the retired patients giving less favorable ratings. However, the interaction between diagnoses and social status offered an alternate perspective for RA and PsA patients. In these groups, working patients provided the lowest ratings, followed by retirees, with disability pensioners giving the highest ratings. At the same time, the axSpA patients followed the trend of the main effects, with the lowest ratings coming from retirees. These findings suggest that patient education and care should aim to incorporate not only the specifics of the diagnosis but also certain demographic characteristics of the patients, such as their social status [34,50,51,52,53]. However, we need to acknowledge that effect sizes were small for all significant terms (diagnosis, social status, and diagnosis by social status interaction), so their practical significance should be interpreted with caution.

In the survey, we also addressed patients’ preparedness to recognize adverse reactions to the bDMARDs treatment and seek timely assistance and advice from respective healthcare providers. In our sample, over 95% of all patients, regardless of the diagnosis, were informed about the potential adverse reactions to the treatment. The most trusted professionals for obtaining assistance in the event of side effects were rheumatologists, followed by nurses, with general practitioners being the least preferred option. Notably, the axSpA group showed the least preference for nursing advice in the case of adverse reactions [54].

The different behavior of the axSpA, which was consistent throughout the findings, raises several questions about the possible reasons leading to their overall less affirmative estimations of nurses’ contributions to disease and treatment informedness, education in healthy living habits, and overall quality of care. Whether this variation is a random occurrence or whether it is due to factors related to the specifics of the diagnosis and treatment procedures, leading to lesser nursing involvement, should be the focus of future research. Qualitative studies could also help identify specific educational needs across different diagnostic groups.

Nursing interventions for patients with rheumatic diseases are diverse and multifaceted but also specific to the patient’s particular diagnosis. They focus on the educational, functional, and psychosocial aspects of the disease. These interventions include educating patients about disease management and medication adherence, supporting self-care strategies, coordinating multidisciplinary care, and monitoring patient outcomes [55,56].

Despite the critical role of nurses’ educational work in treatment adherence and outcomes, organizational metrics such as diagnosis-related groups (DRGs) often undervalue and fail to capture it. Recent research indicates that nursing complexity is linked to DRG weight in hospitalized pediatric patients [57]. This line of research could be expanded to adult patient populations by focusing on the organizational impact of nurse-led education.

### Limitations

Interpretation of our findings should be made in light of several limitations that affect generalizability. Some of them stem from the research instrument, while others are related to the research sample. First, the cross-sectional survey design prevents causal inference, so our explanations are essentially speculative. Second, satisfaction was measured with Likert-type items, which may be influenced by social desirability bias. Treating these ordinal responses as continuous can also contribute to ceiling effects and may reduce sensitivity to group differences. Third, the survey items were developed by the research team; despite efforts to ensure reliability, their validity was not established using standardized instruments. Questions on nurse-led education and training did not reference specific practices or types of support. As a result, some recommendations were extrapolated from overall patterns rather than supported by detailed, item-level data.

Another limitation is that participants were recruited from two rheumatology units in a single Bulgarian city, excluding other healthcare settings. This fact imposes a limitation on the external validity of the study and its generalizability to the role of nurses in the management of IJD throughout the national healthcare system, and it narrows the scope of our recommendations.

## 5. Conclusions

Our findings indicate that nurse-led education in patient self-management skills is highly valued by patients with inflammatory joint diseases. Further developments in specialized training programs tailored to the specific needs of different diagnoses will lead to increased patient satisfaction and a better overall quality of life. Policies should enhance nurse participation in patients with axSpA by tailoring educational materials to their specific needs. Moreover, the development of nurse-led holistic training approaches should also consider the unique needs of patients from diverse demographic backgrounds. Greater investment in specialist rheumatology nursing training could expand the range of services provided and enhance the quality of nurse–patient communication. Investigating nurse-led education could provide further evidence of its organizational benefits and its potential to alleviate patient complexity. Finally, aligning nurse-led interventions with EULAR recommendations may improve the quality of care nationally and provide benchmarks for comparisons with practices across Europe.

## Figures and Tables

**Table 1:** Background information about the patients.

Socio-Demographic Variables	RA *n* = 124	PsA *n* = 64	axSpA *n* = 73	*p*-Value
Age				
○ Median (IQR)	63 (13.0) ^a^	58 (14.5) ^b^	49 (16.5) ^c^	
○ Range	29–80	20–78	25–77	<0.001 ^KW^
Gender *n* (%)				
○ Women	96 (77.4%) ^a^	33 (51.6%) ^b^	21 (28.8%) ^c^	
○ Men	28 (22.6%)	31 (48.4%)	52 (71.2%)	<0.001 ^χ^2^^
Social status				
○ Working	48 (38.7%) ^a^	34 (53.1%) ^a, b^	48 (65.8%) ^b^	
○ Retired	44 (35.5%)	17 (26.6%)	8 (11.0%)	0.001 ^χ^2^^
○ Disability pensioner	32 (25.8%)	13 (20.3%)	17 (23.3%)	
Time since diagnosis (years)				
○ Median (IQR)	13 (11.0)	14 (7.0)	14 (15.5)	
○ Range	4–49	4–32	4–47	0.442 ^KW^
Visits to a rheumatology specialist				
(per-year counts)				
○ Median (IQR)	2 (1.0)	2 (1.0)	2 (0.0)	
○ Range	2–7	2–6	1–6	0.517 ^KW^
bDMARDs treatment *n* (%)				
○ Subcutaneous injection	106 (85.5%)	58 (90.6%)	64 (87.7%)	
○ Intravenous treatment	18 (14.5%)	6 (9.4%)	9 (12.3%)	0.601
Duration of bDMARDs therapy (years)				
○ Median (IQR)	5.50 (4.0)	7 (5.75)	6 (5.0)	
○ Range	1–13	1–12	1–12	0.126 ^KW^

Different superscript letters (a, b, c) denote significant differences between diagnoses at the 0.05 level. KW—Kruskal–Wallis test; ^χ^2^^—Chi-square test.

**Table 2:** Patient ratings of nursing support in enhancing informedness about rheumatic disease and treatment.

In Your Experience, Do Nurses Contribute to Patient Care by Providing Information on the Following Issues?	RA *n* = 124	PsA *n* = 64	axSpA *n* = 73	F-Value	*p*-Value
	Mean (SD)				
The disease and its management	3.60 (0.85)	3.68 (0.78)	3.38 (0.97)	2.33	0.099
The treatment and its specifics	3.60 (0.86)	3.62 (0.87)	3.31 (1.01)	2.67	0.071
The medical examinations and tests	3.66 (0.83)	3.63 (0.85)	3.35 (0.96)	2.91	0.056

Scale: 0—Definitely ‘No’; 1—Rather ‘No’; 2—Neutral; 3—Rather ‘Yes’; 4—Definitely ‘Yes’.

**Table 3:** Confidence in nurses’ competence to deliver training in self-injection of bDMARDs by patients on injectable bDMARDs treatment.

Statistics	RA *n* = 106	PsA *n* = 58	axSpA *n* = 64	F-Value	*p*-Value
EMM	3.90	3.81	3.85		
SE	0.049	0.068	0.064	0.343	0.710

EMM: Estimated marginal mean after controlling for the confounding effect of the variable ‘specialist who trained the patients to self-inject bDMARDs; SE: Standard error; Scale: 0—Definitely ‘No’; 1—Rather ‘No’; 2—Neutral; 3—Rather ‘Yes’; 4—Definitely ‘Yes’.

**Table 4:** Nurses’ role in educating patients to adopt healthy living habits and maintain psychological well-being.

In Your Experience, Do Nurses Contribute to Patient Care by:	RA	PsA	axSpA	F-Value df (2, 258)	*p*-Value
	Mean (SD)				
Providing training aimed at healthy eating, alcohol and tobacco abuse, and improved health culture	3.48 (0.88)	3.52 (0.84)	3.31 (1.00)	1.93	0.147
Educating patients about appropriate physical activity	3.50 (0.88)	3.52 (0.84)	3.34 (1.00)	1.46	0.233
Offering psychological and emotional support	3.72 (0.74)	3.77 (0.69)	3.54 (0.81)	1.88	0.154

Scale: 0—Definitely ‘No’; 1—Rather ‘No’; 2—Neutral; 3—Rather ‘Yes’; 4—Definitely ‘Yes’.

**Table 5:** Results from the one-way ANCOVA with dependent variable patients’ ratings of nurses’ contributions to overall quality of care.

Independent Variables		EMM (SE)	F-Value	ANCOVA *p*-Value	Partial Eta Squared	Bonferroni *p*-Value
Diagnosis						
RA		3.78 (0.81)				RA ↔ PsA: 0.837
PsA		3.63 (0.11)	4.044	0.019	0.032	RA ↔ axSpA: 0.015
axSpA		3.34 (012)				PsA ↔ axSpA: 0.255
Social status						
Working		3.55 (0.08)				W ↔ R: 0.959
Retired		3.38 (0.13)	3.63	0.028	0.029	W ↔ DP: 0.256
Disability pensioner		3.81 (0.11)				R ↔ DP: 0.030
Diagnosis * Social status						
RA	Working	3.57 (0.12)				W ↔ R: 0.131
	Retired	3.82 (0.14)				W ↔ DP: 0.028
	Disability pensioners	3.93 (0.15)				R ↔ DP: 0.295
PsA	Working	3.53 (0.14)				W ↔ R: 0.749
	Retired	3.61 (0.20)	2.56	0.039	0.041	W ↔ DP: 0.390
	Disability pensioners	3.74 (0.23)				R ↔ DP: 0.622
axSpA	Working	3.56 (0.13)				W ↔ R: 0.031
	Retired	2.70 (0.28)				W ↔ DP: 0.299
	Disability pensioners	3.76 (0.19)				R ↔ DP: 0.012

EMM—Estimated marginal mean adjusted for the covariates; SE—Standard error. *: interaction.

## Data Availability

The raw data supporting the conclusions of this article will be made available by the authors on request.

## Abbreviations

The following abbreviations are used in this manuscript:

IJD	Inflammatory Joint Disease
bDMARDs	biological Disease Modifying Anti Rheumatic Drugs
RA	Rheumatoid Arthritis
PsA	Psoriatic Arthritis
AxSpA	Axial Spondyloarthritis
ADRs	Adverse Drug Reactions
PROs	Patient Reported Outcomes
NSAIDs	Nonsteroidal Anti-Inflammatory Drugs
MDT	Multidisciplinary Team
